# Supplementary material for: Living with rheumatic fever and rheumatic heart disease in Victoria, Australia: A qualitative study
Source: PLoS Negl Trop Dis. 2024 Aug 30;18(8):e0012038. doi: 10.1371/journal.pntd.0012038 (PMC11392276; doi:10.1371/journal.pntd.0012038)
Supplement: S1 Appendix — (DOCX) [file pntd.0012038.s001.docx]

**Checklist:**

- Everything charged up, turn off iCloud backup on iPad and iPhone,
- Set the two recording apps on the two devices
- If on ZOOM check the MCRI background is on

**Script:**

Talofa, Bula Vinaka (name), my name is Loudeen, I work at Murdoch Children’s Research Institute.

Thank you for joining our study, we hope our Talanoa will help us to make recommendations to make services for you and other patients to be much better.

We would like to have a Talanoa.

- **Talanoa:** I want you to feel comfortable, and please share only what you feel comfortable to tell me.
- Our talanoa is confidential. Only my team will know about our talanoa.
- Our talanoa will take about 45 minutes-but we can go longer.
- Please at any time let me know if you need to stop, or if you want me to repeat the question or ask me to explain anything.
- **3 Sections to our Talanoa.**
- The first section is about: Living with rheumatic fever or rheumatic heart disease
- The second section I would like to ask you about your experiences getting treatment
- The third section I would like to ask you about how health services could work better for you
- **Recording:** To make sure that I don't miss any of your answers, I would like to record the interview. Is that OK?
- Do you have any questions so far?
- Does that all sound OK to you?
- OK, I’ll start recording now.

I’ll ask you about your experiences about living with RhF

# **Section 1: Living with RhF or RHD**

**First, can you just tell me a bit about yourself, like what do you do, do you have any hobbies?**

**(If caregiver) what’s your relationship to (child)?**

**Initial Diagnosis**

What was it like to find out you/your child was diagnosed with RhF or RHD?

**How long ago was that?**

Tell me what it is like living with **RhF/RHD?**

**Had you ever heard of rheumatic fever of rheumatic heart disease before?** How did you find out about it?

**How much do you think you know about RhF/RHD? Would you say that you Know:**

A Lot

A Little

Not much

**Who have you learnt about it from?**

**Is there anything about RhF/RHD that you would like know about but felt too shy to ask anyone?**

**Are there things in your day-to-day activities which you enjoy doing to help you cope with the challenges of RhF/RHD?**

**Are there any activities that you cannot do because of RhF/RHD?**

**What’s the hardest thing about living with RhF/RHD?**

# **Section 2: Services**

## **Appointments**

**Have you/your child received** *echocardiograms and [BPG] Injections? (If participant looks unsure, BPG is the white antibiotic injections)*

How long and how far do you travel to get to your appointment? (travel mode)

Does it cost you much to get there? Do you get help from anywhere/anyone to come to appointments? Do you pay for echocardiogram or injections?

**How do you remember that you have a BPG appointment?**

- Does the clinic send SMS reminder? Do/would having reminders help?
- *Why/why not?*

**Clinical Premises**:

- What is the waiting room like? What things are there that you like/dislike?
- What is the clinical room like? Is it private? Is it clean? Do you feel comfortable being there for your injection or echocardiogram, or not really?

**Admin/Clinical Staff:**

How are the nurses and doctors?

Are they easy to talk to, or not?

Is there anything they could do to make your appointment better?

## **Treatment Injections:**

**Can you tell me why BPG injections might be recommended for people with RhF and RHD?**

**• Can you tell me why the doctors say you need to have these injections?**

**• Are you just getting them because you were told to, or do you think they help you? How do you think they help?**

**What might happen if you missed an injection?**

- **What about if you missed lots of injections?**

**Do you think that the Injections help you stay healthy or not so much?**

- What could happen if you didn’t take them?
- Do you think missing some would be a problem, or not really? What might happen?
- Is there something you do instead?

**Missed Injections:** Have you ever missed having an injection? (did it happen because you missed an appointment/decided not to receive it?)

Why? How often? (did you not feel well? too painful? was concerned about something at the clinic? Etc)

**Normally, what’s the BPG injection like?**

- Is there anything that helps you get though it? How does that work?

*Prompts – family support, nurse manner, pain relief*

**How painful is it usually?**

**After you’ve had your injection – can you tell us what the rest of the day is like for you? How is your sleeping, eating and coping like?**

**(If applicable) – You mentioned that someone else in your family had RF/RHD. Do they get the injections?** What’s that like for them? How are they coping?

**Apart from your RhF medications, are there other medications you/child have to take? What’s that like?**

**Do you need to miss out on things to get treatment for rheumatic fever/rheumatic heart disease, like work or school?** *(treatment includes BPG)*

What sorts of things do you miss out on? Why? Do others in your family miss out?

**How about school? Did you need much time out?**

Are there things your school could have done to have supported you better?

**How much have you been told about heart surgery for your/child’s condition?**

**Tell me how you feel about the possibility of needing to have heart surgery in the future? (does it worry you, do you know what is involved, or not?)**

**Apart from the family you live with, who else knows that you have RhF/RHD? Do people at your school/work know?**

- Is it easy or hard for you to talk about having RhF/RHD? Why/why not?

**Who is supporting you to get through RF/RHD?**

## Can I ask, does your family live together?

## **Changes to Services**

**How do you think things will change as you get older? Will you be taking on more responsibility for getting treatment?** How do you feel about that?

**Is there anything about the way you receive your RhF/RHD treatment that you would like to talk about?**

**How can RhF/RHD services be improved to make it better for you/your child?**

**Thank you. You’ve shared some really important information about living with rheumatic fever/rheumatic heart disease. That has been really helpful.**

We have come to the end of my questions.

- What will happen now is that the recording will get typed up and then it will be included with everybody else’s for the analysis. Would you like to review a copy of the transcript prior to analysis?
  - - If participant looks unsure or asks about it, “Most people don’t look at the transcript, but you are able to, if you want to.”
- When it’s ready, we shall send a summary of the results from this study to your email so you can see them.
- You will receive an email from the study manager Jane Oliver in the next few days which will have the gift card details. If you don’t receive it, please get in touch with her. Jane’s contact information is on the Information Sheet.

Before we finish, do you have any questions for me?

My supervisor will email you a $50 Prezzee gift card to say thanks for your time today.

*Check – do you have email or phone no. for all the participants for Jane to use to send the voucher?*

*If not, write down one for the child (it can be the caregiver’s as long as the child is happy with this_.*

That’s the end of the interview for today. Thank you so much for your time and all of your help. It is really appreciated.
