# Supplementary material for: Living with rheumatic fever and rheumatic heart disease in Victoria, Australia: A qualitative study
Source: PLoS Negl Trop Dis. 2024 Aug 30;18(8):e0012038. doi: 10.1371/journal.pntd.0012038 (PMC11392276; doi:10.1371/journal.pntd.0012038)
Supplement: S2 Appendix — (DOCX) [file pntd.0012038.s002.docx]

**Checklist:**

- Everything charged up, turn off iCloud backup on iPad and iPhone,
- Set the two recording apps going on the two devices
- Click on ZOOM link
- Check the MCRI/UniMelb study background with all the collab. partner logos is on

**Script:**

Hi (name), my name is…. We spoke earlier.

Thank you for agreeing to be interviewed today for the Understanding Rheumatic Fever study. Is this still a good time to speak?

As we discussed when we set up this interview, in this study, we’re interested in hearing about your experience of working with young rheumatic fever/heart disease patients in Victoria, especially Pasifika patients, and possible ways clinical service delivery could be improved.

When I say Pasifika, I mean people with Micronesian, Melanesian or Polynesian ethnicities. For this study we aren’t talking about New Zealanders so much.

We hope this will help us develop recommendations for clinicians and Government to improve services for Pasifika young people with rheumatic fever and rheumatic heart disease.

Before we get started, I just want to highlight some things about the interview.

- All of the interview questions are optional so please do not feel like you need to answer anything you do not want to. You can skip any question - not a problem at all. I will just go onto the next question. You can also stop participating at any time if you want. You don't need to give a reason, it is really just up to you what you want to answer or not.
- Any information you provide is confidential and importantly, your name will not be included in any analysis or reporting. So while the data is re-identifiable, your name is removed and we will use a pseudonym or a participant number.
- There are also no right or wrong answers. Everything is just what you think, so please feel free to answer with whatever you think.
- The interview will take about 45-60 minutes. We can speak longer if you would like, but I'll check in with you before we go over 45 minutes.
- To make sure that I don't miss any of your answers, I would like to record the interview. The recording will then be typed up so we can analyse it. Are you OK for me to record it?
- Do you have any questions so far?
- Are you still OK to proceed with the interview today? I’m recording now.

Thank you.

Please answer these questions specifically considering young Pasifika patients you’ve seen with rheumatic fever and rheumatic heart disease.

If you haven’t worked much with Pasifika, then feel free to speak more about rheumatic fever and rheumatic heart disease patients generally.

**CLINICAL WORK**

Please can you tell me a bit about what you do in your clinical role?

Have you worked with patients who have rheumatic fever or rheumatic heart disease before?

- Can you tell me a bit about what your work with them involved?

Have you worked much with Pasifika rheumatic fever or rheumatic heart disease patients?

*If no, have you worked much with Pasifika patients with other health issues?*

- - How has this gone?
  - What were the biggest challenges you faced?
  - Was there anything which made the job easier?
  - What do you think were the biggest challenges for the patients?
    - How about for their families and caregivers?
  - Did you notice anything that seemed to help?

*(If worked with Pasifika patients and non-Pasifika ARF/RHD patients, ask about both separately)*

When working with Pasifika patients and families, have you observed any cultural factors which it helps to consider when providing clinical are?

*Eg. Family hierarchies and roles, ways to approach conversations that do work/don’t work, respectful approaches…*

Do you feel you have enough support when working with Pasifika rheumatic fever/rheumatic heart disease patients?

*What other support would you like to have? How would this help?*

Do you feel that you have enough information and resources when working with them?

*What else you like to have? How would this help?*

**TREATMENT DELIVERY**

When you work with rheumatic fever/rheumatic heart disease patients, do they come to your clinic, or does something else happen?

- How well would you say this works?

Can you think of ways that it could be made easier for rheumatic fever/rheumatic heart disease patients to be diagnosed and/or receive treatment?

- Particularly Pasifika patients

*(If applicable)* How well would you say the treatment is tolerated by the patients?

- What can make this easier for them?
- How about for their family or caregivers?
- How do the staff feel about providing the treatment? Is there anything which might make it easier for them to do?

Generally speaking, what sorts of things do you think the patients would benefit from?

*Prompts: Appointment reminders, social services, clinical services, home visits, social supports, financial assistance, cultural considerations*

- Does anything currently exist in this space?
- If Yes, how well would you say it is working?
- How should it work?
- How might it be improved?

Have you needed to use any less routine methods to provide clinical care for rheumatic fever/rheumatic heart disease patients?

*e.g. travel to the patient, opportunistic delivery, phone call with caregiver, arrange for the patient to have transport/financial assistance to receive treatment*

**Scenario 1.** Let’s imagine a new way to get BPG becomes available. Patients would only need to get it once every 3 months, but the process would be a bit different.

They would go to the clinic and the nurse would pop a small needle under the skin of the abdomen. This is less painful than the regular injection into the buttock muscle. Next, over the following 15 minutes the nurse would slowly inject 9 vials (around 20 mLs) of BPG into the belly fat. That’s 9-times more BPG than what is given in one go.

It wouldn’t hurt going in, but it would take about 15 minutes. The patient might have a band-aid over the infusion site for the rest of the day. That night, and the next day, it might feel a bit tender so they might want avoid things like playing contact sports.”

• How would you feel about patients getting this every 3 months instead of the usual BPG? Why is that?

• Do you think it sounds better or worse than the usual treatment?

**Scenario 2. “**Now let’s imagine there’s another way to get BPG. This would also be an injection into the belly fat, but this time it would be delivered in a much smaller volume (less than half of what is normally injected) and with a smaller needle. The patient or their caregiver could do it themselves at home. If doing it themselves, the patient would inject into the belly fat. Or, if someone else was doing it, they would inject into the skin of buttock. They would be taught how to do it at the clinic.

Patients would need this injection once per week. They could do it anytime. Because of the small needle and amount, it would be less painful than the current injection. The injections would come in boxes of 10, so a box would last 2.5 months and they’d need to be kept in the home fridge.

• How would you feel about patients doing this once per week instead of the usual BPG? Why is that?

• Do you think it sounds better or worse than the usual treatment?

• Do you think it would be better for the patients to pick the boxes of injections up from the clinic and have a check in with the staff to see how they’re going, or have it posted out to them directly? Why?

**SYSTEMS**

What sorts of systems do you use in your work with rheumatic fever/rheumatic heart disease patients?

Prompt: Case ascertainment/registration/recall systems

How well would you say these systems work?

*Consider timeliness, data completeness, accuracy, analysis, reporting*

*Is duplication a problem (eg. if entering data into multiple systems)?*

*Do you think the patients find this approach acceptable?*

- Can you think of possible ways these systems could be improved? How might this work?

Let’s say an app becomes available which sends patients reminders about when the next injection was due. They could also look at the app to see when the last dose was and when the next one is due.

• Do you think they would want to use an app like that or not so much? Why?

• What sorts of things do you think would be useful on a patient RHD app?

*Prompts – e.g. information about what to expect when getting BPG, training videos on giving BPG at home (if this became available), information about ARF/RHD, something else?*

• Do you think an app like this would be helpful for clinicians treating ARF/RHD patients, or not so much?

**RESPONSIBILITY**

Once a treatment is clinically recommended for a child with rheumatic fever/rheumatic heart disease, whose responsibility is it to see that they get it at the right time?

*Prompt: The patient, their caregiver, a clinician (which clinicians?) or someone else?*

- How does this change as the patient grows up?

At the end of the day, who do you think makes the decisions about whether to get the treatment?

*Prompt: The patient, their caregiver or someone else?*

- Is that the case for Pasifika patients generally?
- Does this vary according to the patient age?

**PERCEPTIONS of UNDERSTANDINGS**

How well do you think rheumatic fever/rheumatic heart disease patients and caregivers understand this condition?

- *Do some have better understanding that others? What is behind this?*
- *What aspects they seem to understand well and not so well? Why is this?*
- *How about for Pasifika?*

Do you think they understand why the injections are recommended?

*Generally and for Pasifika patients*

- Do you think they usually take this seriously or not so much?

Are you aware of Pasifika patients using alternative therapies much? What are these?

*Eg traditional medicines, spiritual approaches*

- Do you think the alternative therapies complement the recommend treatment, or are replacing it?
  - What might this mean for the patient?

How well do you think clinicians understand the needs of young Pasifika rheumatic fever/rheumatic heart disease patients and their families?

- What do they need to know when working with this group
- What could be done to improve their understandings?

Have you noticed any approaches that clinicians can use which work well for engaging this group?

- What are the best ways for clinicians to gain trust and build rapport?
- What facilitates this?
- What inhibits this? *(eg. staff turnover, tight clinical schedules)*

How well do you think policymakers understand the needs of young Pasifika rheumatic fever/rheumatic heart disease patients and their families?

- What do they need to know when working with this group
- What could be done to improve their understanding?

What do you think would be the best way for young Pasifika rheumatic fever/rheumatic heart disease patients to get the care they need?

- What changes are needed the most to improve their health outcomes?

**Thank you. You’ve shared some really important information about rheumatic fever/rheumatic heart disease. That has been really helpful.**

We have come to the end of my questions.

- What will happen now is that the recording will get typed up and then it will be included with everybody else’s for the analysis. Would you like to review a copy of the transcript prior to analysis?
  - - If participant looks unsure or asks about it, “Most people don’t look at the transcript, but you are able to, if you want to.”
- When it’s ready, we shall send a summary of the results from this study to your email so you can see them.
- Can I offer you a $50 gift card as a thanks for your time today?

You will receive an email from the study manager in the next two weeks which will have the gift card details. If you don’t receive it, please get in touch. The contact information is on the Information Sheet.

Before we finish, do you have any questions for me?

That’s the end of the interview for today. Thank you so much for your time and all of your help. It is really appreciated.
