# Supplementary material for: Living with rheumatic fever and rheumatic heart disease in Victoria, Australia: A qualitative study
Source: PLoS Negl Trop Dis. 2024 Aug 30;18(8):e0012038. doi: 10.1371/journal.pntd.0012038 (PMC11392276; doi:10.1371/journal.pntd.0012038)
Supplement: S4 Appendix — (DOCX) [file pntd.0012038.s004.docx]

**YOUTH FOCUS GROUP AGENDA – Understanding rheumatic fever study**

**Introduction**

Welcome everyone, I’m really pleased to me talking with you again. Thank you so much for taking the time to join us tonight.

- Acknowledgement of country,

*I acknowledge that we are on the lands of the Wurundjeri people, and I pay my respects to them and their elders, past and present.*

- Acknowledge participants taking time to join and discuss ARF/RHD, acknowledge their bravery coming forward to share their experiences and opinions, and hope this will lead to things changing for the better.
- We ask that you please,
- Keep your camera on the whole time
- If possible sit in a quiet place where you can talk without being interrupted
- Don’t be afraid to say what you think and respond to what other people in the focus group say.

**What we’re doing today**

We’re having a group discussion about possible ways to improve things for people with ARF/RHD in Victoria.

Before we start, I just want to remind you that

- You can say as much or as little as you want, there’s no pressure to answer the questions.
- We won’t mention anything specific from your interview, but you can bring up anything you want to.
- It’s a confidential session. We won’t share identifying information that you give us, like names.
- Please respect each other’s privacy and don’t share information about who took part today with anyone else.
- We’re just asking about your opinions and experiences, and there are no right or wrong answers.
- All opinions are welcome. You don’t all need to agree, but all opinions will be heard and respected.
- The information you share will be used to shape recommendations for the government, doctors and nurses, about how their services can be provided better.
- You don’t have to take part today, and you can leave at any time. Nothing bad will happen if you do that.
- This session will run for about a 90 minutes.
- It will be recorded so we don’t miss anything.
- Please use the chat function to send Jane your email or mobile ph no. She will send you a gift voucher as thanks for taking part.
- Any questions before we start? Are you all okay to begin? *(Loudeen start Zoom recording, Jane backup recording)*

Let’s go around and **introduce ourselves,** Jane and I will start.

- Please tell us your name, and what you do – are you working or studying? And your favourite hobby. *1^st^ Loudeen, Jane,* *More confident participant to begin (maybe Sean).*
- Summer is coming! even though it doesn’t feel like it much now. What are you most looking forward to this summer? *(everyone speaks)*
- Now can I ask you to **share what condition you have, RF or RHD**, how old you were when you first had it, and how you’re managing it now, like with pills, injections, or watchful waiting?

**For the next questions, even if you’re not getting injections now, please think back to when you were.**

- Imagine you could change one thing about the injections. What would you change?
- Have you found some people give the injection better than others?

*What do they do that’s different?*

- Is there someone you’d prefer to give the injection to you?
  - - What about when you were younger?
- Have you ever had the **injections overseas or interstate**, outside Victoria?
  - - What was that like compared to here? *Easier/Harder, better/worse? Why?*
    - What did overseas/interstate do well that VIC could learn from?
- If you needed to travel, you think you would be able to get injections interstate or overseas?
  - - How would you find out?
- Hands up, who has had a **dental appointment**?
- Does the dentist know you’ve had rheumatic fever?
  - Do you see the dentist often? Why/why not?
  - Have you taken extra antibiotics before a dental appointment?
  - Do you think the dentist understands rheumatic fever?
  - Would you ask a dentist questions about rheumatic fever?
- How do you feel about **other people in the community knowing** you had rheumatic fever?

*Such as your friends, teachers, boss, family, colleagues/other students?*

- - Is there anything that could make you feel better about them knowing?

**Now let’s talk abut getting older and taking on more responsibility for your health**

In Australia, people are often considered to be adult when they turn 18. Your parents usually make healthcare decisions for you until you are 18.

- How do you feel about that? Do you think 18 is the right age to make your own healthcare decisions, like when to see the doctor?
- How easy or difficult do you think it’ll be to organise your treatment for RF/RHD when you’re 18+?

*Consider… getting to appointments, finding time, wanting/not wanting to go*

- - How about paying for injections and transport? Do you think that will be an issue?
- Do you want your family to keep being involved in your healthcare as you get older, or would you like a bit of space?

*Do you want them coming to your appointments and speaking with your doctors?*

- - Have there been times when you’d prefer to talk to the doctor privately? How did that go?

**Let’s talk about hospital appointments**

- How do you feel about going to hospital for echocardiograms (the heart scan with electrodes) and check-ups?

*Is it important to go, or not really? Why?*

*Is visiting the hospital… fun?*

When patients get older, their hospital appointments might be moved from the Royal Children’s to another hospital, like Footscray or Sunshine.

- How do you feel about going to a hospital you haven’t been to before?
- Is there anything that could make going to a hospital appointment better?

*Would having a friend or family come with you help?*

- Would it be better having visited once with family before having to go there as an adult?

**Ways to get antibiotics**

Now I’d like to run some ideas past you, and get your thoughts on whether these ideas could work for young people with rheumatic fever, or not. Please remember these are just ideas.

Let’s IMAGINE the injection could be **given by a school nurse**. The student with rheumatic fever could pop in to the nurse’s office during a break. The teacher might need to know that they’re seeing the nurse, but the other students wouldn’t be told.

- Do you think people would like to get the injection at school, or not really? *Why? What kinds of things might be going through their mind?*

For older people with rheumatic fever who work, let’s IMAGINE **a nurse could travel to their workplace** and do the injection there.

- Do you think they would like that? Why? What kinds of things might they be thinking?

Do you think it would be better or worse to have the **nurse come to the house** to give injections than to get them at the clinic? *Why?*

**SCENARIOS**

**Scenario 1**. “Let’s IMAGINE a new way to get BPG becomes available. You would only need to get it once every 3 months, 4-times a year, but the process would be a bit different.

You would go to the clinic, lie down, and the nurse would pop a small needle under the skin in your abdomen, just beside your belly button. Next, the nurse would slowly inject around 20 mLs of BPG. That’s 9-times more BPG than what you usually get in one go.

It wouldn’t really hurt going in, but it would take about 15 minutes. Afterwards you’d go home as usual. That night, and the next day, it might feel a bit tender, and you might want avoid things like contact sports.”

• How would you feel about getting this every 3 months instead of the usual BPG? Why is that?

*Do you think it sounds better or worse than the usual monthly injections?*

**Scenario 2. “**Now let’s IMAGINE there’s another way to get BPG. This would also be an injection in the belly, but this time you could use a much smaller amount (less than half of what is normally injected in one go) and use a smaller needle. You could do it yourself at home, or someone in your family could do it. You/they would be taught how to do it at the clinic.

You would need to have this injection once a week at a time that suits you. Because of the small needle and amount, it would be less painful than the usual injection. The injections would come in boxes of 10, so a box would last 2.5 months and would need to be kept in your fridge.

I’m going to **share my screen** and show you what the injections look like”

**(SHOWCARD 1)**

• How would you feel about getting this once a week instead of your usual BPG? Why is that?

*Do you think it sounds better or worse than the usual treatment?*

• Would you feel okay about injecting yourself in the abdomen, or would you rather inject it somewhere else? *Where?*

• Would you feel okay about having it injected in your ventrogluteal area, the same place as where the current injection is given, by someone in your family, or not so much?

Now let’s IMAGINE you could pop a cover over the injection, like this. **(Share screen - SHOWCARD 2)** To inject it, you would click the button on the top like this.

• Do you think this cover would make home injections better, worse or just the same? Why?

• Would you prefer to pick the boxes of 10 injections up from the clinic and have a check in with the staff to see how you’re going, or would you prefer to have it posted out to you? Why?

- If you needed it, would you prefer to have the antibiotics injected at home weekly or 3 monthly in the clinic like we described?

**Considering something else now; a Support Group**

- How would you feel about a RF/RHD support group? If one existed, would you like to go along and meet other people who had had rheumatic fever?
- Do you think Mum and Dad would like to go along?
- If we’re planning a rheumatic fever support group, what kinds of things should we keep in mind?

*Who would be able to join? Where would you like it to be held? What would you like / not like about it?*

**Thank you so much for your time today!**

We’ve talked about lots of ideas for making treatment better, but now I’d like to ask you if you have any **ideas of your own** that you would like to share?

*It can be anything at all*

**Thank you all so much** for sharing your thoughts today. It has been extremely helpful, and valuable for our research.

- Are there any questions or comments before we go?

We really appreciate your time today.

- If you haven’t already, please send Jane your mobile phone no. or email so she can send you your voucher.

Thank you and bye!
