# Supplementary material for: Living with rheumatic fever and rheumatic heart disease in Victoria, Australia: A qualitative study
Source: PLoS Negl Trop Dis. 2024 Aug 30;18(8):e0012038. doi: 10.1371/journal.pntd.0012038 (PMC11392276; doi:10.1371/journal.pntd.0012038)
Supplement: Attachment — Submitted filename: Response to reviewers_PLOS-NTDs (1).docx [file pntd.0012038.s006.docx]

**Living with rheumatic fever and rheumatic heart disease in Victoria, Australia: a qualitative study**

We would like to thank the peer reviewers and editors at PLOS Neglected Tropical Diseases for the time they spent considering our submission. Our response to each comment is detailed as follows.

| **Reviewer 1** | | |
| --- | --- | --- |
| **Comment** | **Response** | **Location of change** |
| Three of the four interview guides include questions about how people would respond to different ways of administering BPG but there was nothing about this in the results.  Will these results be the subject of a separate paper? | These questions were not actually asked in the parent/child interviews, only in the focus group.  This decision was made so as to spend adequate time to ensure participants understood the proposed interventions.  These results will not be the subject of another paper. The findings are summarised in lines 345-348 and in the recommendation to support the development of technologies that make secondary prophylaxis more convenient and less painful. | Parent/child interview guide |
| Another bias is that the recruitment strategy is likely to have biased towards over-selection of metropolitan patients. Based on the notification data available since July 2023, what proportion of patients live outside the metropolitan area and how are their experiences likely to differ from those of study participants? | As of today’s date, 24 cases have been notified - only 6 have the local government area (LGA) reported. All reported LGAs are in, or bordering, metropolitan Melbourne.  As RCH is the major quaternary public paediatric hospital, and all suspected paediatric RF/RHD cases are recommended to be referred there for assessment, it is unlikely that the sampling strategy is overly biased towards detecting metro cases. <https://www.rhdaustralia.org.au/victoria>  Consequently we have not commented further in the Discussion, but have added contextual information to the Introduction. | Introduction lines 95-98 |
| Given that "All patient participants granted the researchers permission to access their RCH medical record" was any attempt made to triangulate self- and parent- reported adherence with BPG secondary prophylaxis against BPG injections documented in the medical record? There might be social desirability bias in reporting high adherence and this part of the results is a bit discordant with the barriers reported. | BPG injection uptake is not documented in the RCH medical records. Given the absence of a Victorian register, it is not possible to assess adherence beyond requesting PBS data, which is beyond the scope of this study. Contextual information has been added to the Introduction.  The possible impact of response bias is mentioned in the Discussion. | Introduction, line 92. |
| Typo in line 434 - " ethically-matched" should probably be replaced by " ethnically-matched" | Corrected | Discussion, line 458 |
| Although this study focused on children, many of the issues raised are likely to be as, if not more, relevant for adult Pasifika people with RHD living in Australia as many are on agricultural work visas, in rural/remote areas with limited primary health care and do not have access to Medicare or PBS-funded medications. | Comment added in the Discussion | Discussion, lines 439-442. |

Very minor edits to have been made enhance the accuracy of the text, detailed below.

| **Correction** | **Location of change** |
| --- | --- |
| In June 2023, ARF and RHD became notifiable in Victoria. | Abstract, line 21 |
| 21-28 days | Introduction, line 81 |
| …identified through by a paediatrician who was associated with the clinics reviewing RCH records. | Methods, line 152 |
| Dollar amount removed | Methods, line 196 |
| if *S. pyogenes* was detected. | Results, line 280 |
| …translated to a control program, should a nurse…. | Discussion line 460 |
